# Time-Serial Evaluation of the Development and Treatment of Myopia in Mice Eyes Using OCT and ZEMAX

**DOI:** 10.3390/diagnostics13030379

**Published:** 2023-01-19

**Authors:** Xueqing Ding, Jinzhen Tan, Jing Meng, Yilei Shao, Meixiao Shen, Cuixia Dai

**Affiliations:** 1 College of Science, Shanghai Institute of Technology, Shanghai 201418, China; 2 College of Computer Science, Qufu Normal University, Qufu 276825, China; 3 School of Ophthalmology and Optometry, Wenzhou Medical University, Wenzhou 325035, China

**Keywords:** optical coherence tomography, ZEMAX, myopia, form-deprivation myopia

## Abstract

Myopia is a significant cause of visual impairment which may lead to many complications. However, the understanding of the mechanisms of myopia is still limited. In this paper, in order to investigate the development and the treatment of myopia, we analyzed the biological structure parameters of mice eyes, obtained from optical coherence tomography (OCT), and the optical performance of mice eyes calculated using ZEMAX software (ZEMAX Development Corporation, Kirkland, WA, USA) in which the optical model was built on the segment-by-segment optically corrected OCT 3D-images. Time-serial evaluation of three groups of mice eyes (form-deprivation myopia mice eyes, normal mice eyes, and atropine-treated myopia mice eyes) was performed. In addition to the biological structure parameters, imaging performance with the development of root-mean-square wavefront aberration at six filed angles was compared and analyzed. Results show that the biological structure parameters of the eye are closely related to the development of myopia. The peripheral defocus of the retina has a significant impact on inducing myopia, which verifies the new theory of myopia development. The delaying effect of atropine solution on myopia development is shown to verify the therapeutic effect of the medicine. This study provides technical support for the investigation of the myopia mechanism.

## 1. Introduction

The increase of myopia on a global scale is becoming a serious health hazard [1,2]. Patients with severe myopia have a high risk of chorioretinal abnormalities such as retinal detachment, cataract, chorioretinal atrophy, and lacquer crack [3,4]. Though the harm of myopia and its associated ocular complications are generally known, the exact molecular mechanism of myopia has not been completely revealed. It is essential to improve the understanding of the mechanisms of myopia, detect early degenerative diseases, and to further study effective methods to control the progression of myopia [5].

In order to detect myopia and investigate its pathogenesis, the imaging techniques of magnetic resonance imaging (MRI), ultrasonic biomicroscopy system, and optical coherence tomography (OCT) have been clinically used to study changes in parameters of biological structure [6,7,8], which are considered to be highly related to myopia. Among these detection technologies, MRI and ultrasonic biomicroscopy can be used to obtain the curvature and thickness of the intermediate layers of human eyes. However, they are limited by resolution [6], and the samples imaged by ultrasonic biomicroscopy must be immersed in the couplant in which the contact examination has the risk of infection or corneal damage [7]. Different from these methods, OCT is a non-contact, non-destructive imaging technique that acquires depth-resolved images of the eyes and has the advantages of nano axial resolution and fast imaging. For the prevention and treatment of ophthalmic diseases such as myopia and cataract, studies on measuring the biological parameters of the eye by OCT has been deeply investigated [8,9]. It has been profoundly demonstrated that OCT has great potential for micrometer-scale biometry as a quantitative imaging device for calculating the biological parameters of myopic eyes.

However, most quantitative morphological measurements are based on the raw OCT images which are distorted by several mechanisms such as the refraction of probe light in the sample and the scan geometries. The calculation of the raw OCT images is inevitably inaccurate. For analysis of ocular pathological changes caused by eye diseases which will affect the shape and dimensions of all structures in an eye, efforts should be firstly made to reduce or compensate for these distortions [10,11,12]. Several recent studies have identified sources of distortions in the generation of OCT image study procedures for reducing the effects of distortions. Methods have been successively proposed to correct the distortion in the anterior-segment-of-eyes OCT images induced by the refraction of probe light in tissue and that of retinal images caused by fan-scanning geometries, respectively [13,14]. Recently, we proposed a segment-by-segment optical correction method for whole-eye OCT imaging which reduced the image errors accumulating over the depth in a stack of different layers of eye tissues. The accuracy of this mathematical correction model was verified in experiments on ex vivo phantom and in vivo experiments of mouse and human eyes [15]. Based on the whole-eye corrected OCT images, in this study, we accurately measured the biological structure parameters of the mice eyes to study the morphological change in the process of myopia.

In addition to the changes of the biological parameters, the aberrant optical performance of the refractive system of eyes is suggested to be a key factor in inducing myopia. A hypothesis about myopia holds that high axial aberration or specific patterns of peripheral refraction may speed up the development of myopia [16,17,18,19]. Many studies are interested in the effect of the optical performance on myopia and a good many meaningful results have been obtained [20,21,22]. In the conventional myopia study, the optical performance of the refractive system of eyes is measured using techniques such as the spatially resolved refractometer or the retinoscopy with low resolution [23,24]. In recent years, ZEMAX software, which is traditionally used to design and analyze optical systems, has been proposed and applied as a powerful tool for the evaluation of the image quality of the refractive system of eyes [25,26,27]. Since the optical model of eyes used in the ZEMAX is established using biological parameters which are measured imprecisely with the instrument at low resolution, the analysis of eye imaging quality is still not accurate enough. In this study, based on the optical model developed on the corrected 3D whole-eye images which were verified in human and mice eyes, the performance of the eye optical system was calculated to deduce the relationship between myopia and the optical characteristic of eyes. To the best of our knowledge, this is the first study on the optical performance of the refractive system of eyes simultaneously using the corrected 3D whole-eye OCT images and ZEMAX software.

In this study, eyes of three groups of mice (form-deprivation myopia mice eyes, normal mice eyes, and atropine-treated myopia mice eyes) were time-serially imaged in 4 weeks by our lab-built Spectral Domain (SD)-OCT system. Original OCT images with light distortion were corrected interface by interface for whole-eye segments from cornea to retina. Data of the axial biological parameters of the mouse eyes were calculated and the three-dimensional expansion polynomial coefficients of each refractive interface were imported into ZEMAX software to establish a ray-tracing model to obtain the optical performance of the mice eyes. By tracking the changes process of the axial biological parameters of the mice eyes and the imaging performance of central and peripheral retina in the development and treatment of myopia, we deeply explored the pathogenesis of myopia and the control mode of atropine solution on myopia.

## 2. Materials and Methods

### 2.1. Subjects

Since ocular changes associated with experimental myopia in mice have typical features of primate myopia, mice are always used as animal models for studying the pathogenesis of myopia [28]. In this study, thirty mice of the same weight, sex, and age (23 days, C57BL/6) were used which were provided by Shanghai Jiesijie Biotechnology Co., Ltd. (Shanghai, China), and all procedures adhered to the provisions approved by the animal ethics committee of Wenzhou Medical University.

Firstly, thirty mice were randomly divided into three groups for comparative experiments. The first group of mice was the myopia model group: ten mice wore a monocular occluder in their left eyes for 4 weeks to induce form-deprivation myopia (FDM), and their right eyes remained untreated [29,30]. The second group was the normal control group: ten mice grew normally in the same laboratory breeding environment. The third group of ten mice was the atropine treatment group, which was established to study the therapeutic effect of the atropine solution on myopia. The necks of all mice wearing eye masks were equipped with plastic collars to prevent the mice from taking out the occluder.

In the first two weeks, mice in the atropine treatment group were treated the same as those in the myopia model group to induce myopia. From the end of the second week, the eye mask of the left eye of mice in the atropine treatment group was removed every morning by applying 50 mg atropine sulfate eye drops with a concentration of 0.01% onto the left eye for 10 min and then the eye mask was put back onto the eye. During the whole treatment process, all right eyes were kept untreated.

### 2.2. Spectral Domain Optical Coherence Tomography

**Figure 1:**
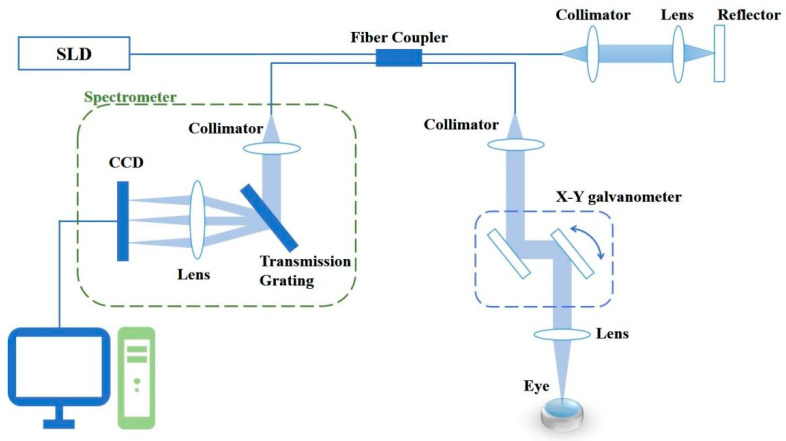
Schematic diagram of SD-OCT system.

In this study, mice eyes were imaged using a lab-built SD-OCT system. The schematic of the SD-OCT system is shown in Figure 1. A super light-emitting diode (SLD, Superlum, BLM2-D-860-G-5) light source with a central wavelength of 860 nm and a bandwidth of 80 nm was used. The beam from the light source passed through the 50:50 optical fiber coupler and was divided into two beams, which entered the sample arm and the reference arm, respectively. In the sample arm, the collimated light was focused on the target sample by an X-Y galvanometer scanner (Thorlabs, GVS002) and a scanning objective lens (Thorlabs, AC254-100-B-ML). In the detection arms, the reflected beams from the sample and reference arms were collimated and detected by a spectrometer which consisted of an 1800 line/mm transmission grating, a multi-element imaging lens (f = 150 mm), and a line-scan CCD camera (EV71YE4CL2010-BA9, 2048 pixels with 10 microns pixel size, e2V). The spectrum information was then transmitted to the image acquisition device (NI PCIe-1433) and transferred to a computer for signal processing and image display. In OCT imaging, we performed 256 A-lines in each B-scan and scanned 256 discrete B-scan positions to produce a volumetric map of eye imaging. The final image data volume of the OCT was 256 × 256 × 2048 pixels *x* × *y* × *z*, and the *x*-axis and *y*-axis scanning range was 4.4 mm with a maximum imaging depth of 5.8 mm. The longitudinal resolution of the imaging system was 6.4 μm and the lateral resolution was 15.9 μm. In this study, the imaging speed was set to 30 kHz.

### 2.3. Correction of Distortions in OCT Images

The originally reconstructed OCT images are affected by artifacts caused by several mechanisms such as the refraction of probe light in the sample and the scan geometries [31,32]. In this study, the whole-eye OCT imaging of mice eyes was performed. All structures of the mice eye could be shown in one B-scan image in which refractions occurred at all the tissue boundaries responsible for the image distortion. Our recent study presented a systematic segment-by-segment correction method to realize light-distortion correction from cornea to retina without any boundary fitting. Comparative experiments with other optical correction methods on mice and human eyes demonstrated the highest accuracy of the proposed method to recover OCT images to their real physical scale [13,14,15].

The process of the segment-by-segment light-distortion correction is divided into four steps. Firstly, the image contrast was enhanced by histogram equalization, and the speckle noise was reduced by median filtering. The image was intercepted according to the width of retina, leaving the region of interest (ROI) easy to calculate; then, based on Fermat’s principle and Snell’s law, the probing light at each interface (air, cornea, lens, vitreous, and retina) was refracted sequentially. Finally, the exact boundaries were deducted and defined in the corrected OCT image. On this basis, the thickness, curvature radius, and axial length of the original and corrected images could be precisely calculated.

**Figure 2:**
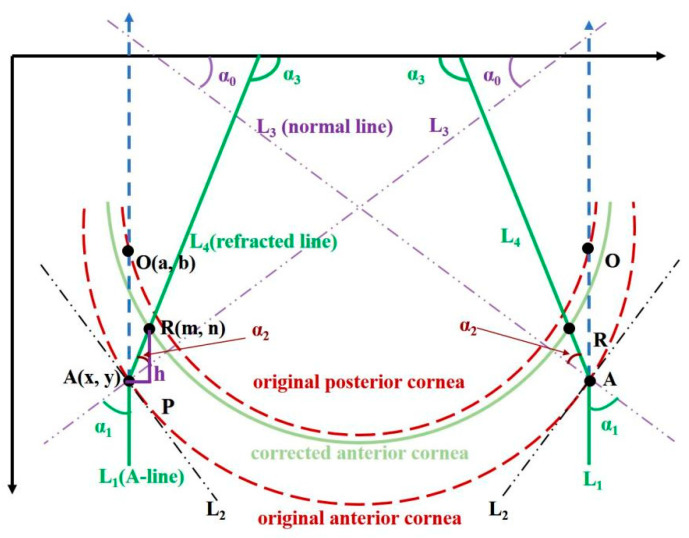
Schematic example of refractive-index correction of air–cornea interface.

Here, we take the light-distortion correction in the air–cornea interface as an example to illustrate the process which is shown in Figure 2. $L_{1},\;L_{2},\;L_{3,}\;and\;L_{4}$ are the incident light (overlapping with A-line in air), tangent line of the anterior corneal surface, normal line of anterior corneal surface, and the refracted light, respectively, O(a, b) is the intersection point of $L_{1}$ between the posterior surfaces of the cornea, and $\alpha_{0}$ represents the angle between the normal and the *x*-axis; $\alpha_{1}$ and $\alpha_{2}$ is the incident angle and refraction angle.

As can be seen from Figure 2, $\alpha_{2}$ can be calculated from Snell’s Law:

(1) $$\alpha_{0}+\alpha_{1}=\pi /2$$

(2) $$k_{1}\;*\;sin\alpha_{1}=k_{2}\;*\;sin\alpha_{2}$$

$k_{1}$ in formula (2) is the refractive index of air and $k_{2}$ is the refractive index of cornea.

Based on the inverse ray-tracing theory, we calculated the path of the refracted light and corrected the position of point O(a, b) at the posterior boundary in the original OCT dataset to its actual position R(m, n).

Setting the length between point O(a, b) and point A(x, y) to be the parameter d, the length h of the refracted light AR after correction was calculated according to Fermat’s theorem:

(3) d=y−b,

(4) $$h=k_{1}d/k_{2}$$

The right triangle ΔARP as shown in Figure 2 was constructed with AR as the hypotenuse. Letting the slope of AR be $K_{AR}$, the coordinate value of the point R(m,n) was calculated by the following formula:

(5) $$m=x-h\;*\;sign\left(K_{AR}\right)cos\left(\alpha_{0}+\alpha_{2}\right),$$

(6) $$n=y-h\;*\;cos\left(\alpha_{0}+\alpha_{2}\right),$$

Regarding the corrected light between the anterior and posterior surfaces of the cornea as the incident light of the light path on the posterior surface of the cornea, $\alpha_{3}$ was the incidence angle of the corrected light on the real posterior surface of the cornea. Continuing to repeat the above calculation steps, the light path in all intraocular tissues was corrected for each boundary. Details can be seen in reference [15]. In the correction process, the refractive index parameters at 840 nm incident beam used for eye tissues were: cornea 1.4015, aqueous humor 1.3336, lens 1.557, and vitreous 1.3329 [33,34,35,36], respectively.

**Figure 3:**
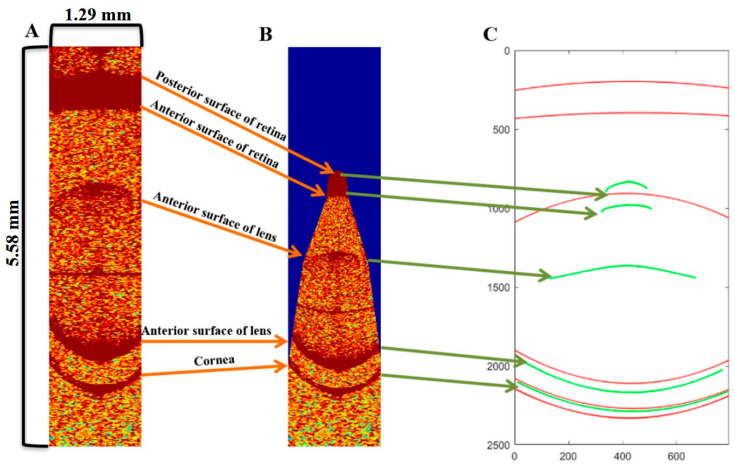
The correction process of mice eyes: (**A**) OCT images with distortions; (**B**) Rectified OCT images; (**C**) The fitted boundary curves of images in ((**A**), marked in red) and after correction ((**B**), marked in green).

The correction effect of the segment-by-segment refractive-index correction on the whole-eye OCT image of mice eye is shown in Figure 3. Figure 3A shows the region of interest preprocessed in the original OCT image which represents the refractive error; Figure 3B is an effect diagram after the after light-distortion correction of Figure 3A; Figure 3C shows the fitted boundary curve of each refractive interface of OCT images, in which the red lines represent the fitting curve before correction (Figure 3A) and the green lines represent the fitting curve after correction (Figure 3B). The biological parameters of mice eyes were then calculated from Figure 3C, and they were further used as the data source of the optical tracking model with the ZEMAX software.

### 2.4. Establishment of Optical Model

**Figure 4:**
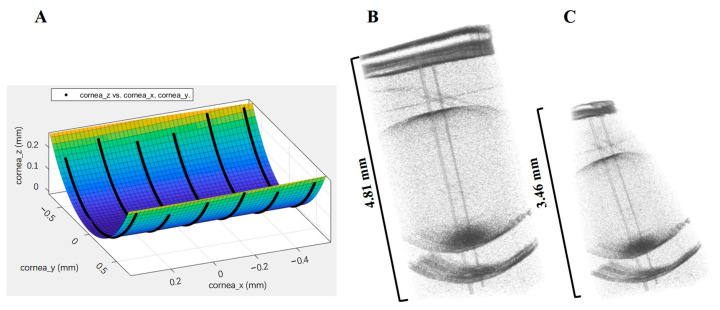
Schematic diagram of the fitting result: (**A**) The fitting function of the anterior surface of the cornea; (**B**) Stereograms of the whole eye before correction; (**C**) Results of the correction of B.

After obtaining the accurately corrected OCT images, in order to fit the real structure of mice eyes, we used MATLAB software to calculate the three-dimensional fitting surfaces of each refractive interface (cornea, lens, vitreous, retina). Among the 256 B-scan images, seven equally spaced images with clear boundaries (serial numbers as 97, 107, 177, 127, 137, 147, and 157) were selected to extract the three-dimensional coordinates of each refractive fitting curve. After the calculation using the Curve Fitting Tool of MATLAB, the extended polynomial coefficients of the three-dimensional surface of each biological structure can be obtained. Figure 4 shows schematic diagrams of the fitting results of the anterior segment of cornea (Figure 4A), stereograms of the whole eye before correction (Figure 4B), and the result of correction (Figure 4C). The coordinate system in the Figure 4A is a three-dimensional coordinate system with the corneal vertex as the origin and the *z*-axis direction as the visual axis direction.

**Figure 5:**
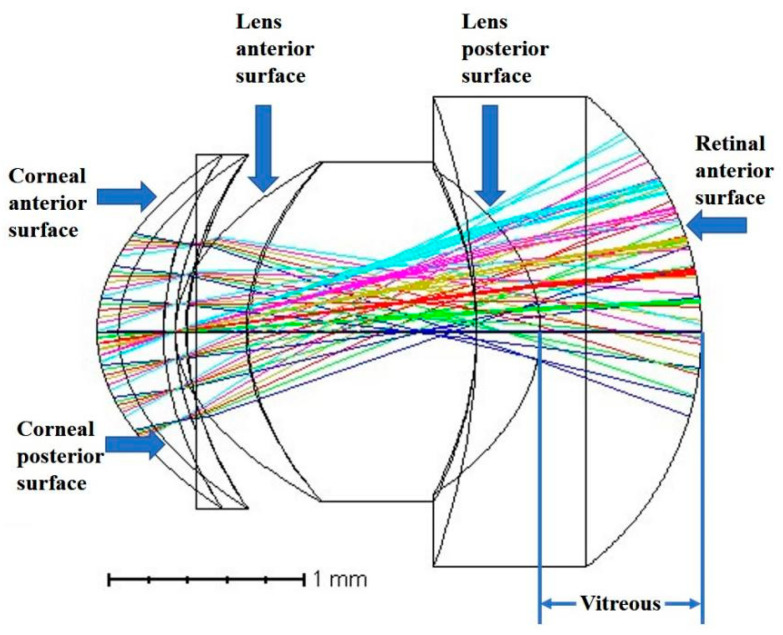
The ray-tracing model in ZEMAX software.

Combining the biological parameters of each mice eye and the coefficients of the extended polynomial of the fitting surface with the refractive index data of mice eye tissue, the optical reflective system of mice eyes was imported into the ZEMAX software. The refractive index data used by the model was consistent with the correction process. The ray-tracing model established by ZEMAX is shown in Figure 5, where the incident wavelengths were set to the visible light series wavelengths preset by the software (0.655 and 0.450). Six different colors of light shown in Figure 5 represent six different fields of view angles of incident light (0°, 5°, 10°, 15°, 20°, and 25°). The optical system parameters such as aberration, wavefront diagram, and point spread function of the mice eye model can be analyzed by ZEMAX. In order to analyze the correlation between the data to investigate the mechanisms of myopia, the root mean square (RMS) wavefront aberration of the model calculated from ZEMAX was used.

## 3. Results

### 3.1. Time-Serial Evaluation of Ocular Biological Parameters

**Figure 6:**
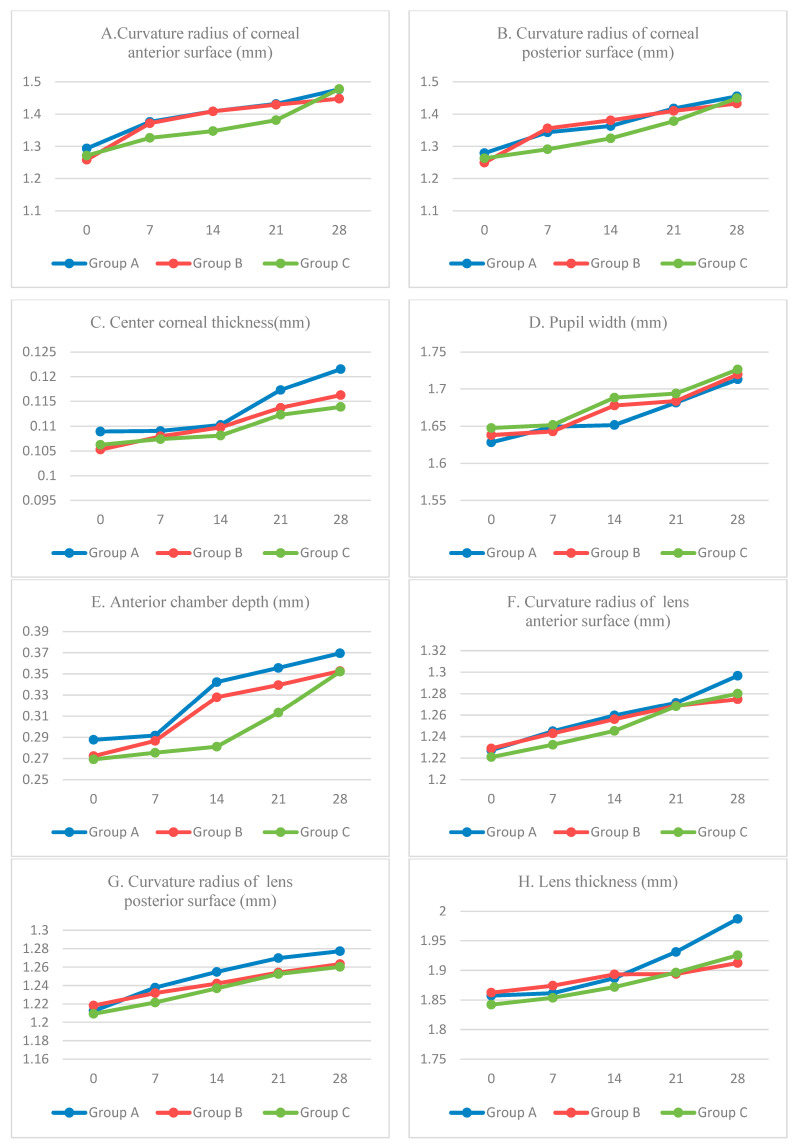

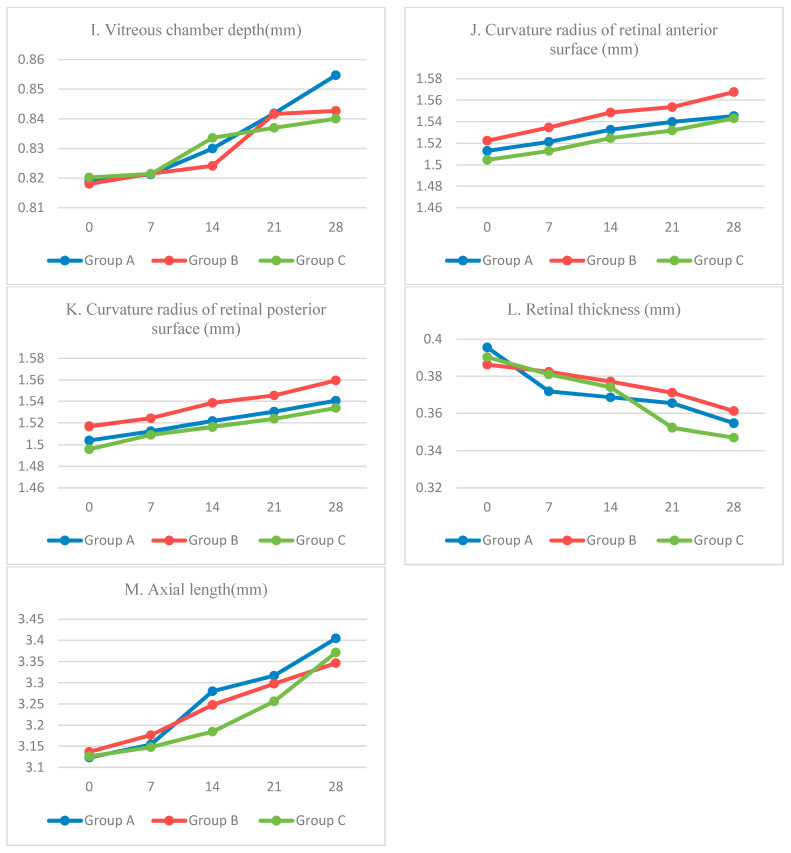
Time-serial development of biological structure parameters. Group A: FDM myopia model group; Group B: normal control group; Group C: atropine treatment group.

In this study, thirteen biological parameters of the left eyes (the thickness of cornea, lens, and retina, and the radius of curvature of their anterior and posterior surfaces, eye axis length, anterior chamber depth, vitreous chamber depth, and pupil width) in three groups of mice were measured once a week. Since the experimental term of this study is 28 days, five evaluation experiments were performed. Results are as shown in Figure 6, in which changes of mice eye structural parameters are compared and analyzed.

In Figure 6, thirteen sub-graphs are provided according to the sequence of light propagation sequence in the eyes. Group A, Group B, and Group C represent the data of the left eye of mice in FDM myopia model group, normal control group, and atropine treatment group, respectively. The legends 0, 7, 14, 21, and 28 in each picture represent the date of the experiment.

As shown in Table 1, changes of these parameters of the form-deprivation myopia group, normal group, and atropine treatment group are obviously observed. The data in Table 1 shows the D-value of thirteen eye biological parameters of three groups of mice before and after the experiment.

The eye changes of three groups of mice can be seen in Figure 6 and Table 1. During the experiment, the axial parameters of the mice of Group A, including axial length, corneal thickness, anterior chamber depth, and vitreous chamber depth had increased, and the degree of growth of these parameters in Group A are significantly larger than those in Group B, which is obviously caused by the form-deprivation myopia. The changes of mice of Group B are caused by the increasing of age and a natural degree of myopia, but the D-value of eye parameters is much smaller than that caused by form-deprivation myopia. In addition, the changing trend of these parameters is similar to the reported studies about myopia mice [35,36,37,38,39,40,41,42,43,44].

Compared with the parameters of the curvature radius of normal mice eye in Group B, the D-value of the corneal and retinal curvature radius of Group A is smaller; meanwhile, the surface of the cornea and retina becomes steeper, and the D-value of the curvature radius of the lens of Group A is larger than Group B. The surface of the lens of Group A is flatter than Group B. These changes are probably due to the self-regulation of the eye to compensate for axial myopia [37].

After atropine treatment, the changing trend of eye parameters in Group C was smaller than that in Group A, which confirms that the atropine solution is effective in the treatment of myopia.

### 3.2. Time-Serial Evaluation of Imaging Performance

**Figure 7:**
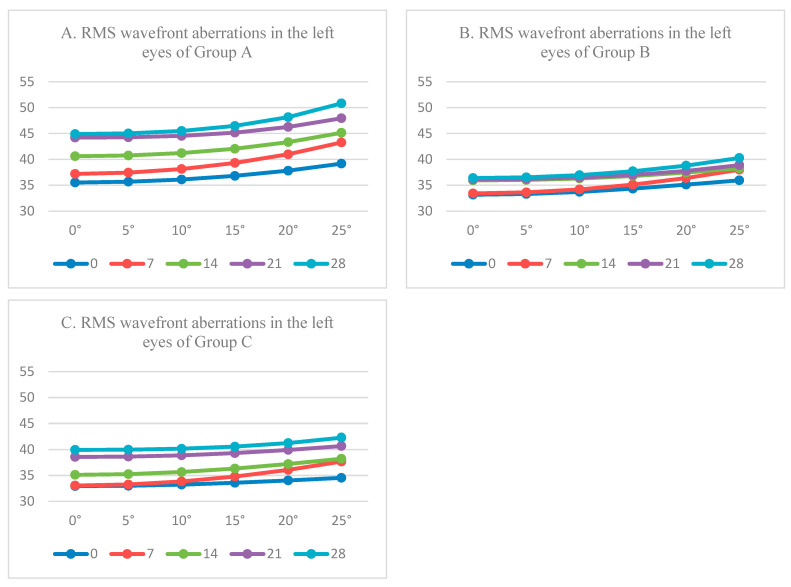
The RMS wavefront aberrations of mice.

After the biological parameters of eye structure and three-dimensional fitting polynomial coefficients were imported into ZEMAX software to establish a ray-tracing model, changes of imaging performance of the left eyes of the three groups were calculated. In Figure 7, the RMS wavefront aberration (polyhedral wavelength) of the mice eyes refractive system under six different field angles was analyzed, in which the icon label represented the experimental days. It can be observed from Figure 7 that the RMS wavefront aberrations (0, 5°, 10°, 15°, 20°, and 25°) of the left eye of mice from Group A increased by 9.3720, 9.3499, 9.3857, 9.6594, 10.3433, and 11.6646 µm; the RMS of mice from Group B decreased by 3.2270, 3.2227, 3.2464, 3.3670, 3.6740, and 4.3234 µm; and the growth of Group C was 6.9970, 6.9827, 6.9455, 6.9904, 7.2256, and 7.7815 µm. It can be seen that the increase in RMS wavefront aberration in myopia mice eyes is significantly larger than that in Group B, and the imaging quality is significantly worse. The RMS wavefront aberration of myopia treated with atropine is obviously smaller than Group A, which indicates that the atropine solution has obviously played a certain therapeutic effect on myopia treatment.

### 3.3. Statistics Correlation Analysis

The correlation between changes of eye parameters and optical performance with myopia development was further studied using SPSS software on the myopia model group. Table 2 shows the correlation between thirteen parameters and the RMS wavefront aberration under six different field angles. Thirteen letters A to M are used to replace thirteen biological parameters in Table 2. The data given in the tables are the significant results of Pearson correlation analysis (*p*-value, Pearson correlation test). A significant correlation is shown when the *p*-value of the two parameters is less than 0.05, and “*” is marked to the data in Table 2. The correlation of the two parameters is significant at the 0.01 level if the *p*-value less than 0.01, and two “*” is added to the data in Table 2.

According to the correlation between RMS wavefront aberration at different field angles and thirteen biological parameters in Table 2, it can be found that thirteen parameters are significantly correlated with RMS wavefront aberration from 0° to 20° (p ≤ 0.05). Among them, six parameters are significantly correlated at the level of 0.01 with the RMS at angle 0° (p ≤ 0.01); seven parameters are significantly correlated at the level of 0.01 with the RMS of 5° and 15°; and eight parameters are highly correlated at the 0.01 level with the RMS of 10° and 20°. Only one parameter is significantly correlated with RMS wavefront aberration at angle 25° (p ≤ 0.05). Axial length and curvature radius of cornea, lens, and retina are significantly correlated with wavefront aberration at the level of 0.01, while the correlation between other parameters and RMS wavefront aberration is significantly correlated at the level of 0.05, which indicates that the overall length of the axis of the eye and curvature radius of the flexor surface had more significant effects on wavefront aberration.

The significant correlation between the changes of biological parameters of the mouse eye and RMS wavefront aberration suggests that these parameters play an important role in the development of myopia. RMS wavefront aberration with a larger field angle is more significantly correlated with the biological parameters of mouse eyes; it can be seen that RMS wavefront aberration of the peripheral retina is more relevant to biological structural parameters than those of central retina which indicates that peripheral defocus may have a greater effect on myopia.

## 4. Discussion and Conclusions

In order to explore the mechanism and treatment of myopia, the time-serial study of the biological structural parameters of mice eyes (form-deprivation myopia mice eyes, normal mice eyes, and atropine-treated myopia mice eyes) was performed. The biological structural parameters and imaging performance of three groups of eyes were measured using OCT and ZEMAX ray-tracing technology. Correlation between biological structural parameters and correlation between biological structural parameters and RMS wavefront aberration of mice eyes in different field angles (0, 5, 10, 15, 20, and 25 degrees) were analyzed. The results showed that the changes of biological structural parameters of mice eyes were closely related to the development of myopia. RMS wavefront aberration of the eye’s peripheral field angle played a more critical role in the development of myopia than the RMS wavefront aberration of the central field angle. The atropine solution has a good therapeutic effect on myopia.

In this study, wide-angle OCT performance was limited by the diameter of the pupil in the raster scanning geometries. Using the dual-channel, dual-focus whole-eye imaging system, which we built in 2012, wide-field OCT images could be obtained. Abundant information in a much wider peripheral field angle could be achieved which would improve the study of myopia. Moreover, in addition to the atropine solution used in this study, more treatments of myopia could be tried and monitored by using this technique for further study.

In conclusion, compared with many other types of research on myopia, this study of both biological structural parameters and optical performance gave a more comprehensive understanding for the mechanism of myopia. Since measurement of the biological structural parameters of mice eyes was based on corrected OCT images which were recovered to their real physical scale, the accuracy of the calculation was fundamentally ensured. The powerful function of ZEMAX and the optical model built on the corrected 3D OCT images guarantee the precision of optical performance detection. Therefore, this study provides a useful technical support for the investigation of the mechanism and treatment of myopia.

## Figures and Tables

**Table 1:** The D-value of eye parameters before and after the experiment.

Eye Parameters	Group A	Group B	Group C
Curvature radius of corneal anterior surface (mm)	0.18314	0.18906	0.20604
Curvature radius of corneal posterior surface (mm)	0.17591	0.18326	0.18600
Center corneal thickness (mm)	0.01261	0.01092	0.00758
Pupil width (mm)	0.08467	0.08166	0.07890
Anterior chamber depth (mm)	0.08189	0.08047	0.08295
Curvature radius of lens anterior surface (mm)	0.06936	0.04579	0.05918
Curvature radius of lens posterior surface (mm)	0.06474	0.04494	0.05091
Central lens thickness (mm)	0.12945	0.04989	0.08313
Vitreous chamber depth (mm)	0.03546	0.02466	0.01982
Curvature radius of retinal anterior surface (mm)	0.03227	0.04508	0.03862
Curvature radius of retinal posterior surface (mm)	0.03661	0.04259	0.03800
Retinal thickness (mm)	−0.04092	−0.02511	−0.04328
Axial length (mm)	0.28114	0.20966	0.24514

**Table 2:** Correlations between the RMS wavefront aberration and biological parameters.

	A	B	C	D	E	F	G	H	I	J	K	L	M
0°	0.018 *	0.007 **	0.030 *	0.024 *	0.005 **	0.011 *	0.004 **	0.024 *	0.012 *	0.001 **	0.003 **	0.050 *	0.000 **
5°	0.014 *	0.005 **	0.030 *	0.021 *	0.005 **	0.009 **	0.003 **	0.023 *	0.011 *	0.001 **	0.002 **	0.042 *	0.000 **
10°	0.008 **	0.002 **	0.031 *	0.016 *	0.007 **	0.005 **	0.002 **	0.022 *	0.011 *	0.000 **	0.001 **	0.030 *	0.000 **
15°	0.004 **	0.001 **	0.032 *	0.011 *	0.012 *	0.003 **	0.001 **	0.021 *	0.011 *	0.000 **	0.001 **	0.018 **	0.002 **
20°	0.002 **	0.000 **	0.035 *	0.007 **	0.022 *	0.001 **	0.002 **	0.021 *	0.013 *	0.002 **	0.001 **	0.010 **	0.008 **
25°	0.052	0.112	0.416	0.247	0.183	0.157	0.063	0.356	0.300	0.109	0.153	0.029 *	0.160

* If the *p*-value of the two parameters is less than 0.05, it can be considered that there is a significant correlation between them, and an “*” is added after the data; the correlation of the two parameters is significant at the 0.01 level if the *p*-value is less than 0.01, and two “*” is added to the data [45].

## Data Availability

Data is contained within the article. The data presented in this study are available in pictures and tables within this article.
